# Chronic alcohol consumption exacerbates murine cytomegalovirus infection via impairing nonspecific and specific NK activation in mice

**DOI:** 10.1096/fba.1019

**Published:** 2018-11-26

**Authors:** Alex Little, Yuanfei Li, Faya Zhang, Hui Zhang

**Affiliations:** ^1^ Department of Pharmaceutical Sciences College of Pharmacy and Pharmaceutical Sciences, Washington State University Spokane Washington; ^2^ Department of Oncology The First Hospital of Shanxi Medical University Taiyuan China

**Keywords:** alcohol, cytomegalovirus, NK cells

## Abstract

Chronic alcohol consumption increases the susceptibility to infectious diseases by compromising the immune system. Cytomegalovirus infection is common in humans and usually is asymptomatic in immunocompetent people. However, it can induce life‐threatening medical complications in immunocompromised individuals such as alcoholics. How chronic alcohol consumption exacerbates cytomegalovirus infection is not known. Herein, we used a mouse cytomegalovirus model to study the underlying cellular and molecular mechanism. We found that alcohol consumption increased viral titers in spleen after 4 days of infection, enhanced body weight loss and inhibited splenomegaly during the acute phase of infection. Blood level of IFN‐β, splenic IFN‐γ and granzyme B‐producing NK cells were lower in alcohol‐consuming mice than in water‐drinking mice at 12 hours after viral infection. Moreover, alcohol consumption decreased IL‐15‐producing DC after 36 hours infection, inhibited NK cell, specifically Ly49H^+^ NK cell maturation and proliferation 3‐6 days after viral infection. Surprisingly, alcohol consumption enhanced NK cell and CD8^+^ T‐cell continuous activation and increased granzyme B‐producing cells. However, alcohol consumption decreased the expression of perforin in spleen and liver. Taken together, chronic alcohol consumption exacerbates cytomegalovirus infection via impairing nonspecific and specific NK cell activation, specifically IFN‐γ and perforin production.

## 1 INTRODUCTION

Cytomegalovirus (CMV) is a member of the herpesvirus family and has the key characteristics of this viral family: species specificity, latency, and reactivation.^1^, ^2^ CMV infection is very common in humans. According to the statistics from the Centers for Disease Control and Prevention (CDC), more than half of adults by age 40 have been infected with CMV in the United States. In most cases, CMV infection is asymptomatic for immunocompetent people. However, CMV infection can induce life‐threatening medical complications in immunodeficient individuals such as organ transplant patients.^3^, ^4^ Because of the clinical significance, the pathogenesis and immunology of CMV infection have been studied extensively.^1^, ^5^, ^6^ Murine cytomegalovirus (MCMV), a homologue of human CMV, is a natural mouse pathogen sharing many functional, genomic, and pathogenic similarities with human CMV.^7^, ^8^ MCMV is an ideal animal model to study CMV biology, pathology, and antiviral immune response.

CMV can infect multiple types of organs such as spleen, liver, lung, and salivary glands. Spleen and liver are the two organs that are rapidly infected and most severely damaged after CMV infection.^9^ Multiple types of cytokines and immune cells are involved in the anti‐CMV immune response and among those IL‐12, IFN‐α/β, IFN‐γ, and IL‐15 are the most important cytokines with NK cells and CD8^+^ T cells being important immune effector cells that control viral replication and clearance.^10^, ^11^, ^12^, ^13^, ^14^ NK cells play a pivotal role in the control of CMV infection. The NK cell response determines the magnitude of early CD8^+^ T‐cell anti‐CMV response. Thus, a stronger NK cell response will lead to a weaker CD8^+^ T‐cell response and memory formation.^15^, ^16^ Mouse NK cells develop a specific C‐type lectin receptor, Ly49H, to recognize the MCMV‐derived glycoprotein m157.^17^ The expression of Ly49H on mouse NK cells determines the resistance of the mouse to MCMV. C57BL/6 mice are resistant to MCMV infection because this strain of mice possesses Ly49H^+^ NK cells.^18^ The lack of Ly49H^+^ NK cell leads to the high susceptibility and severe MCMV infections in strains like BALB/c mice.^19^ The specific recognition between Ly49H and m157 allows innate lymphocyte NK cells to generate immunological memory function after MCMV infection, which is the hallmark usually only processed by adaptive immune cells.^20^

Based on the activation status of NK and CD8^+^ T cells, the antiviral immune response during the acute phase of MCMV infection can be divided into three stages. The first stage is the nonspecific NK cell activation stage.^21^ At the early stage of acute MCMV infection, viral infection stimulates stromal cells in the spleen and liver to produce type I interferon, IFN‐α/β, which in turn activates NK cells, including both Ly49H^+^ and Ly49H^‐^ NK cells, to produce IFN‐γ (type II interferon).^22^ The type I and type II interferons are key to controlling the first round of MCMV replication in spleen and liver, which completes around 28‐32 hours after MCMV infection.^22^ Because all of the NK cells are activated regardless Ly49H status, this stage is designated as the nonspecific NK cell activation stage.^21^ The second stage is Ly49H‐specific NK cell activation stage. With the completion of the first round of viral replication and dissemination of MCMV to the surrounding cells, the expression of m157 in the virally infected cells activates Ly49H^+^ NK cells proliferation, maturation and cytotoxicity to kill virally infected cells. This stage is dominated by Ly49H^+^ NK cell activation and is called the Ly49H‐specific NK cell activation stage.^21^ Three days after viral infection, CD8^+^ T cell become activated and start to clear virally infected cells. Therefore, the last stage is the CD8^+^ T‐cell activation stage.

It is well known that chronic alcohol consumption decreases the number and impairs the cytotoxicity of NK cells and CD8^+^ T cells in human and experimental animals.^23^, ^24^, ^25^, ^26^ Alcohol consumption increases the susceptibility to infectious diseases such as pneumonia and HCV infection.^27^, ^28^ It is also expected that alcohol consumption increases the risk of CMV infection and reactivation. Indeed, clinical case reports indicate that alcohol consumption increases the risk of co‐infection of CMV in pneumonia and HIV^+^ patients and worsens the progression of these diseases.^29^, ^30^ CMV infection in alcoholics leads to life‐threatening liver decompensation.^31^ Research in experimental animal models also indicates that alcohol consumption worsens liver injury and delays viral clearance.^32^, ^33^ However, the precise mechanism of how alcohol consumption affects anti‐MCMV immune response remains to be elucidated. Herein, we used a chronic alcohol consumption mouse model to study how alcohol consumption affects the dynamic changes of cytokines, NK and CD8^+^ T‐cell response during an acute phase of MCMV infection. We found that alcohol consumption decreases the production of IFN‐β, IL‐12, and IL‐15 at the early stage of viral infection, impairs nonspecific and Ly49H‐specific NK cell activation and CD8^+^ T‐cell response. The outcome is impaired viral clearance and enhanced body weight loss.

## 2 MATERIAL AND METHODS

### 2.1 Animals and alcohol administration

Female C57BL/6 mice at 6‐7 weeks of age were purchased from Envigo (Indianapolis, IN) and housed in WSU‐Spokane PBS vivarium, which is fully accredited by the Association for the Assessment and Accreditation of Laboratory Animal Care. Mice were housed in plastic cages with micro‐filter tops and CareFresh beddings and allowed free access to Purina 5001 rodent laboratory chow and sterilized Milli‐Q water. After 1 week of acclimation, mice were randomly divided into two groups. One group was continuously provided with chow and sterilized Milli‐Q water as a control. The other group was provided with chow and 20% w/v alcohol diluted from 190‐proof Everclear (St. Louis, MO) with sterilized Milli‐Q water. Mice consume at least 30% of their caloric intake from alcohol. The daily intake of alcohol was 5.6 ± 1.0 mL/d. The blood alcohol concentration was about 0.03%.^34^ Mice were fed with alcohol for 3 months before further experiments. This is the time period where the changes induced in immune parameters by alcohol consumption is stable.^35^ The animal protocol was approved by the Institutional Animal Care and Use Committee at Washington State University.

### 2.2 MCMV infection, body weight measurement, and viral titration

Smith strain MCMV was a generous gift from Dr Lewis L. Lanier (University of California, San Francisco). The virus was propagated in 4‐week‐old female BALB/c mice and was prepared from salivary glands after 21 days of viral infection. After 3 months of alcohol consumption, C57BL/6 mice were infected with 1 × 10^5^ pfu/mouse of MCMV in 200 µL of PBS via intraperitoneal injection. Alcohol‐consuming mice were continuously treated with 20% alcohol until they were euthanized at the indicated time points after viral infection. Body weight was measured before viral infection (Day 0) and measured daily after infection. Body weight loss was calculated by the following formula: Body weight loss = (Body weight in day X − Body weight in day 0)/Body weight in day 0 × 100. Viral titers were determined by standard plaque assay with mouse embryonic fibroblast cells (MEF, from Lanier lab, University of California, San Francisco).^36^

### 2.3 Antibodies and reagents

The following FITC‐, PE‐, APC‐, PE‐Cy5.5‐, PE‐Cy7‐, PE‐eFluor 610‐ labeled anti‐mouse antibodies were purchased from eBiosciences (San Diego, CA) or BioLegend (San Diego, CA): anti‐CD3 (145‐2C11), anti‐CD4 (GK1.5), anti‐CD8 (53‐6.7), anti‐CD44 (IM7), anti‐CD69 (H1.2F3), anti‐CD11b (M1/70), anti‐CD27 (LG.3A10), anti‐CD127 (A7R34), anti‐KLRG1 (2F1), anti‐NK1.1 (PK136), anti‐Granzyme B (NGZB), anti‐Ly49H (3D10), anti‐IFN‐γ (XMG1.2), anti‐TNF‐α (MP6‐XT22), anti‐IFN‐β (MIB‐5E9.1), anti‐IL‐12 (C15.6), anti‐CD16/CD32 (clone 93). Biotin‐labeled anti‐IL‐15 was purchased from R & D systems (Minneapolis, MN). PMA and ionomycin were purchased from Sigma‐Aldrich (St. Louis, MO).

### 2.4 Sample collection and leukocyte isolation

At the indicated time points after viral infection, mice were euthanized. Blood was collected by cardiopuncture. Serum was prepared by centrifugation of the clotted blood. Spleen and livers were collected and the weight was measured. Approximately half of each organ was used for leukocyte isolation. The rest of the organs were stored at −80°C for viral titration assay and for isolation of protein and RNA for western blot and PCR to determine gene expression. The isolation of leukocytes from spleen and liver follow our previously published methods.^37^

### 2.5 Phenotype analysis of leukocytes by flow cytometry

Immunophenotyping of splenic and liver leukocytes was determined by flow cytometry as we reported before.^37^ Briefly, 0.2‐1 × 10^6^ cells were incubated with 5 µL of anti‐mouse CD16/32 (clone 93, BioLegend) in a 96‐well plate on ice for 5 min to block the Fc receptor. Then, cells were mixed with the antibody cocktail containing antibodies for specific cell surface marker staining and incubated on ice for 30 minutes in the dark. Cells were washed twice with FACS buffer (PBS + 0.1% BSA + 0.09% NaN3) and analyzed by Gallios flow cytometer and Kaluza software (Beckman Coulter).

### 2.6 Cytokine intracellular staining

Splenocytes or liver leukocytes were cultured in RPMI 1640 medium supplemented with 10% FBS, 1% penicillin and streptomycin and 5 µg/mL of Brefeldin A in a 24‐well plate at 2 × 10^6^ cells/mL with or without PMA (50 ng/mL) and ionomycin (500 ng/mL). Cells were incubated in a 5% CO_2_ incubator at 37°C for 4 hours. Then, cells were harvested and washed with FACS buffer twice. Cell surface staining was conducted as described above. Then, cells were fixed and permeabilized with BD Cytofix/Cytoperm™ kit for 20 minutes following the manufacturer's instruction. Cells were washed with Perm/wash buffer twice and incubated with antibodies against specific cytokines on ice for 30 minutes in the dark. Cytokine‐producing cells were analyzed by Gallios flow cytometer and Kaluza software (Beckman Coulter).

### 2.7 Western blot

Liver and spleen samples were prepared by RIPA lysis and extraction buffer supplemented with proteinase and phosphatase inhibitors (Sigma, St. Luis, MO) following the manufacturer's instruction. Proteins were separated by SDS‐PAGE and transferred to a nitrocellulose membrane. The membrane was blocked with 5% BSA blocking buffer for 1 hour and washed with TBST buffer five times. The membrane was incubated with primary antibody at 4°C overnight. After five washes with TBST buffer, the membrane was incubated with HPR‐conjugated secondary antibody at room temperature for 1 hour. The membrane was washed with TBST buffer and then developed with SuperSignal™ West Pico PLUS Chemiluminescent Substrate (ThermoFisher Scientific) and analyzed by Bio‐Rad Gel Documentation System.

### 2.8 Statistical analysis

Data were analyzed by Microsoft Excel and GraphPad Prism. Two‐tailed Student's *t* test or two‐way ANOVA with uncorrected Fisher's LSD test were used to determine the significance of difference between the water‐drinking and alcohol‐consuming mice. The difference was considered significant between the two groups when *P* < 0.05.

## 3 RESULTS

### 3.1 Chronic alcohol consumption increases viral load, inhibits splenomegaly, and enhances body weight loss during acute MCMV infection

**Figure 1:**
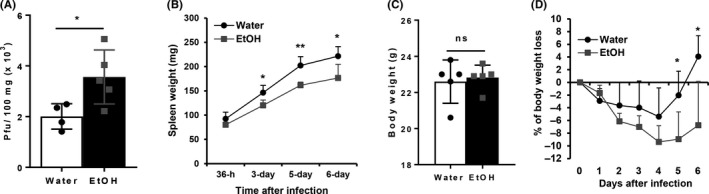
Effects of chronic alcohol consumption on viral clearance, splenomegaly, and body weight loss during the acute phase of MCMV infection. After 3 months of alcohol consumption, mice were infected with 10^5^ pfu of MCMV. A, Viral titers in spleen after 4 days of infection. B, Spleen weight at the indicated time points after viral infection. C, Body weight measured before MCMV infection (day 0). D, Percentage of body weight change at the indicated time points after viral infection compared to the body weight before viral infection (day 0). Data were analyzed by two‐tailed, unpaired Student's *t* test (A, C) or Two‐way ANOVA with Fisher's LSD test. Data = mean ± SD. Each group contained 4‐5 mice in each independent experiment. Results are a representative of at least two biologically independent experiments with similar results. Water = water‐drinking mice. EtOH = alcohol‐consuming mice. *P < 0.05, **P < 0.01. ns, not significant

MCMV replication and dissemination are tightly controlled by the immune system, specifically by NK cells. If alcohol consumption impairs NK cell function as we found previously,^37^ it would increase the viral load during acute MCMV infection. To test this hypothesis, we used plaque assay to determine the viral load in the spleen 4 days post infection (pi). Results indicated that MCMV viral titers in the spleen of alcohol‐consuming mice were significantly higher than in the spleen of water‐drinking mice (Figure 1A).

MCMV infection induces extramedullary hematopoiesis (EMH), that is, viral infection‐activated blood formation at the fetal hematopoiesis organs, such as spleen. Splenomegaly, which is induced by EMH, is one of the hallmarks of MCMV infection. NK cells play a key role in the induction of splenomegaly.^38^ We next determined if alcohol consumption affects splenomegaly during acute MCMV infection. Results indicated that the spleen weight of alcohol‐consuming mice was significantly lower than the spleen weight of water‐drinking mice from 3 to 6 days pi (Figure 1B).

MCMV infection also induces significant body weight loss. The magnitude of body weight loss is correlated with severity of viral infection.^39^ We next determined how alcohol consumption affects body weight change during the acute phase of MCMV infection. In this chronic alcohol consumption model, alcohol consumption does not affect body weight.^40^ Body weight was measured before MCMV infection (day 0). There was no difference in the average body weight between the two groups of mice (Figure 1C). Body weight loss was observed in both groups of mice after MCMV infection (Figure 1D). In water‐drinking mice, the body weight only on day 4 pi was statistically significantly lower than the body weight on day 0 (*P* < 0.05). These mice recovered their body weight on day 5 pi and gained some weight on day 6 pi (Figure 1D). The weight loss of alcohol‐consuming mice was more significant. The weight from day 2 pi through 6 pi was significantly lower than the weight on day 0 (*P* < 0.05) (Figure 1D). Body weight loss reached the maximum value on day 4 pi, and did not recover on day 6 pi The body weight of alcohol‐consuming mice was significantly lower than their water‐drinking counterparts on day 5 and 6 pi (Figure 1D).

These results indicate that chronic alcohol consumption impairs viral clearance, inhibits splenomegaly, enhances body weight loss and delays body weight recovery during the acute phase of MCMV infection.

### 3.2 Chronic alcohol consumption impairs nonspecific NK cell activation at 12 hours after MCMV infection

**Figure 2:**
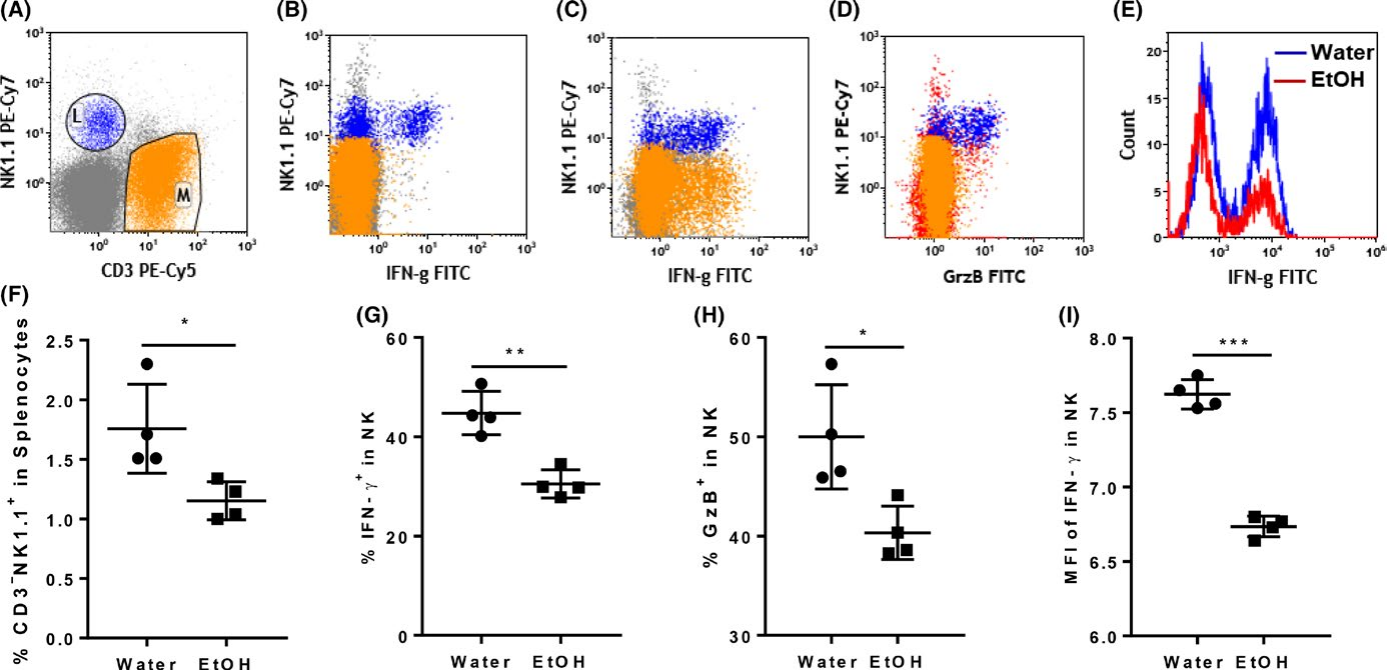
Effects of chronic alcohol consumption on IFN‐γ and granzyme B production of splenic NK cells after 12 hours MCMV infection. After 12 hours MCMV infection, mice were euthanized. Splenocytes were isolated and cultured with or without PMA+ ionomycin. IFN‐γ and GzB producing NK cells were determined by intracellular staining and flow cytometry. A, dot plot shows electronic gate on NK cells (L) and T cells (M). B, Dot plot shows that without PMA+ ionomycin stimulation NK cells are the only cell population producing IFN‐γ. C, Dot plot shows with PMA+ ionomycin stimulation both T cell and NK cells produced IFN‐γ. D, Dot plot shows that without PMA+ ionomycin stimulation NK cells were the only cell population that produced GzB. E, Histograms show IFN‐γ producing NK cells in the spleen of water‐drinking mice and alcohol‐consuming mice. F, Percentage of CD3^‐^NK1.1^+^ NK cells in splenocytes. G, Percentage of IFN‐γ‐producing cells in splenic NK cells without PMA+ ionomycin stimulation. H, Percentage of GzB‐producing cells in splenocytes without PMA+ ionomycin stimulation. I, Mean fluorescence intensity (MFI) of IFN‐γ in splenic NK cells without PMA+ ionomycin stimulation. Data were analyzed by two‐tailed, unpaired Student's *t* test. Data = mean ± SD. Each dot or square stands for one individual mouse. Each group contained 4‐5 mice in each independent experiment. Results are a representative of at least two biologically independent experiments with similar results. Water = water‐drinking mice. EtOH = alcohol‐consuming mice. *P < 0.05, **P < 0.01, ***P < 0.001

NK cell anti‐MCMV immune response can be divided into nonspecific NK cell activation and Ly49H^+^ specific NK cell activation. Cytokine‐induced nonspecific NK cell activation can take place as early as 12 hours pi. IFN‐γ produced by nonspecific NK cell activation plays an important role in the control of the first round of viral replication.^22^ We next used flow cytometry to determine how alcohol consumption affects nonspecific NK cell activation at 12 hours pi. Figure 1A is a dot plot showing CD3^−^NK1.1^+^ NK cells and CD3^+^NK1.1^−^ T cells. Without extra stimulation, only NK cells produced IFN‐γ and GzB (Figure 2B, D). With PMA stimulation, both NK cells and T cells produced IFN‐γ (Figure 2C), suggesting that NK cells are the only cell population that is activated to produce IFN‐γ and GzB at 12 hours pi. At this time point, the percentage of NK cells was lower in the spleens of alcohol‐consuming mice than in their water‐drinking counterparts (Figure 2F). The percentage of splenic IFN‐γ‐ and GzB‐producing NK cells was also lower in alcohol‐consuming mice compared to water‐drinking mice (Figure 2G, 2H). The mean fluorescence intensity (MFI), which stands for the relative amount of protein in each single cell, of IFN‐γ in splenic NK cells was significantly lower in alcohol‐consuming mice than in water‐drinking mice (Figure 2E, I). These results suggest that alcohol consumption impairs nonspecific NK cell activation after MCMV infection.

### 3.3 Chronic alcohol consumption decreases blood level of IFN‐β and IL‐12 at 12 hours after MCMV infection

**Figure 3:**
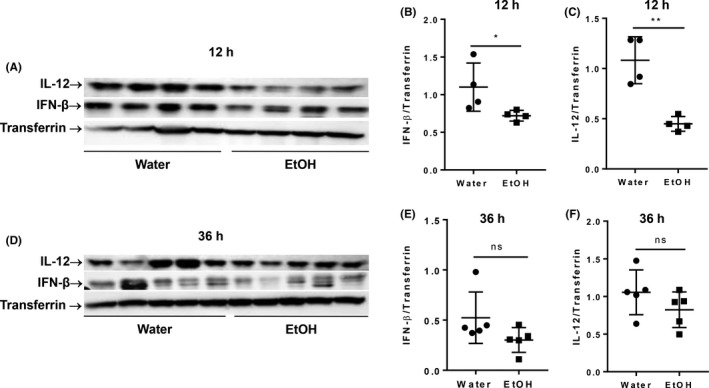
Effects of chronic alcohol consumption on the blood level of IFN‐β and IL‐12 at 12 and 36 hours after viral infection. The expression of IFN‐β and IL‐12 in serum was determined by western blot at the indicated time points. Transferrin was used as a loading control. A. Electrophoretogram shows western blot of IL‐12, IFN‐β and transferrin in serum at 12 hours after MCMV infection. B and C, Ratio of IFN‐β (B) and IL‐12 (C) to transferrin in the serum after 12 hours of MCMV infection. D, Electrophoretogram shows western blot of IL‐12, IFN‐β and transferrin at 36 hours after MCMV infection. E and F, Ratio of IFN‐β (B) and IL‐12 (C) to transferrin in the serum after 36 hours MCMV infection. Data were analyzed by two‐tailed, unpaired Student's *t* test. Data = mean ± SD. Each dot or square stands for one individual mice. Each group contained 4‐5 mice in each independent experiment. Results are a representative of at least two biologically independent experiments with similar results. Water = water‐drinking mice. EtOH = alcohol‐consuming mice. *P < 0.05, **P < 0.01. ns, not significant

Cytokines, specifically type I interferon (IFN‐α/β) and IL‐12, play a key role in nonspecific NK cell activation.^22^ IFN‐α/β levels can be elevated as early as 8 hours pi, and peak at 12 hours pi.^22^ We next determined the blood level of IFN‐β and IL‐12 at 12 and 36 hours pi by western blot. Results indicated that chronic alcohol consumption significantly decreased IFN‐β and IL‐12 in the blood at 12 hours pi (Figure 3A‐C). At 36 hours pi, the blood level of these cytokines were still lower in the alcohol‐consuming mice compared to water‐drinking mice, but it was not statistically significant (Figure 3D‐F).

### 3.4 Chronic alcohol consumption decreases IL‐15‐producing dendritic cells in spleen and liver 36 hours after MCMV infection

**Figure 4:**
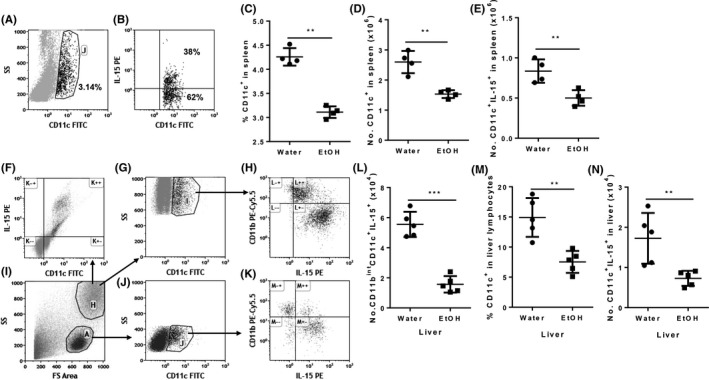
Effects of chronic alcohol consumption on IL‐15‐producing DC in spleen and liver after 36 hours of MCMV infection. A, Dot plot shows CD11c^+^ DC (gate J) in splenocytes. B, Dot plot shows IL‐15‐producing cells (upper right quadrant) in gated CD11c^+^ DC. C, Number of CD11c^+^ cells in the spleen. D, Number of CD11c^+^ cells in the spleen. E, Number of CD11c^+^IL‐15^+^ cells in the spleen. F, Dot plot shows IL‐15 producing cells are CD11c^hi^ cells in liver granular cells. G, Dot plot shows gated CD11c^hi^ DC in liver granular cells. H, Dot shows that CD11c^+^CD11b^int^ cells (right lower quadrant) are the potent IL‐15‐producing cells in liver granular cells. I, Dot plot shows granular cells (gate H) and lymphocyte (gate A) in liver leukocytes. J, Dot plot shows gated CD11c^+^ cells (gate J) in liver lymphocytes. K, Dot plot shows that most of the CD11c^+^CD11b^int^ cells (right lower quadrant) are the potent IL‐15‐producing cells in liver lymphocytes. L, Number of CD11c^+^CD11b^int^IL‐15^+^ cells in liver. M, Percentage of CD11c^+^ cells in liver lymphocytes. N, Number of CD11c^+^IL‐15^+^ lymphocytes in liver. Data were analyzed by two‐tailed, unpaired Student's *t* test. Data = mean ± SD. Each dot or square stands for one individual mice. Each group contained 4‐5 mice in each independent experiment. Results are a representative of at least two biologically independent experiments with similar results. Water = water‐drinking mice. EtOH = alcohol‐consuming mice. *P < 0.05, **P < 0.01, ***P < 0.001

MCMV infection elevates IL‐15 production. IL‐15, especially DC‐produced IL‐15, plays a critical role in NK cell proliferation, maturation and cytotoxicity.^41^, ^42^, ^43^ Therefore, IL‐15 is the key cytokine to control Ly49H^+^ specific NK cell activation during MCMV infection.^44^ We next determined how alcohol consumption affects IL‐15 production at 36 hours pi. IL‐15 is not secreted in a soluble form and instead is expressed as a membrane‐bound cytokine binding to IL‐15Rα on the surface of IL‐15‐producing cells. IL‐15 activates NK and CD8^+^ T cells via trans‐presentation.^45^ We used flow cytometry to determine the IL‐15‐producing DC population. Splenic CD11c^+^ DC and IL‐15‐producing DC in splenic DC are shown in Figure 1A and B, respectively. The percentage and number of splenic CD11c^hi^ DC in alcohol‐consuming mice were significantly lower than in water‐drinking mice (Figure 4C, D). The number of splenic IL‐15‐producing DC in alcohol‐consuming mice was also significantly lower than in water‐drinking mice (Figure 4E). CD11c^hi^ DC in the liver distribute in two populations of cells: granular cells and lymphocytes (Figure 4I). In liver granular cells, CD11c^hi^ DC are the major IL‐15‐producing cells 36 hours pi (Figure 4F). Almost all of these CD11c^hi^ DC produce IL‐15 (Figure 4G, H). Based on the expression of CD11b, these DC can be further divided into CD11b^int^ and CD11b^hi^. IL‐15 expression in CD11b^int^ DC was significantly higher than in CD11b^hi^ DC (Figure 4H). The number of IL‐15^+^CD11b^int^CD11c^hi^ DC was significantly lower in alcohol‐consuming mice than in water‐drinking mice (Figure 4L). Same as granular DC, the liver lymphoid DC can also divided into CD11b^int^ and CD11b^hi^, and IL‐15 expression was higher in CD11b^int^ DC than in CD11b^hi^ DC (Figure 4K). Alcohol consumption significantly decreased the percentage of CD11c^+^ DC and the number of lymphoid IL‐15‐producing DC in the liver at 36 hours pi (Figure 4M, N). Collectively, these results indicate that chronic alcohol consumption decreased IL‐15‐producing DC in the spleen and liver at 36 hours pi.

### 3.5 *Chronic alcohol consumption decreases Ly49H^+^ NK cells at the early stage of MCMV infection*

**Figure 5:**
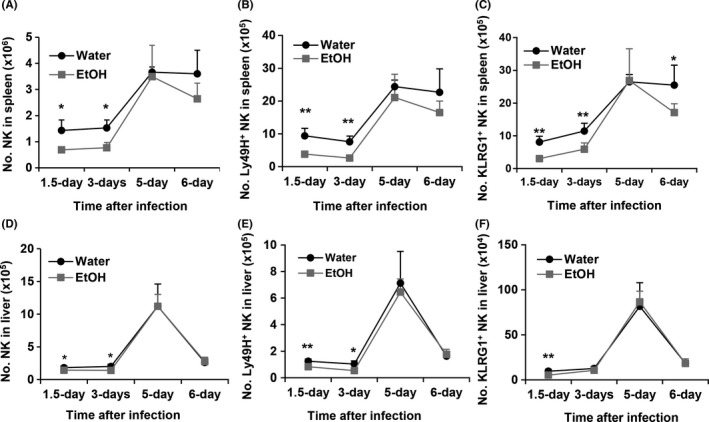
Effects of chronic alcohol consumption on the dynamic changes of NK cells, Ly49H^+^ NK cells and KLRG1^+^ NK cells at the indicated time points in the spleen and liver. A, Number of NK cells in the spleen. B, Number of Ly49H^+^ NK cells in the spleen. C, Number of KLRG1^+^ NK cells in the spleen. D, Number of NK cells in the liver. E, Number of Ly49H^+^ NK cells in the spleen. F, Number of KLRG1^+^ NK cells in the spleen. Data were analyzed by two‐way ANOVA with uncorrected Fisher's LSD test. Data = mean ± SD. Each group contained five mice in each independent experiment. Results are a representative of at least two biologically independent experiments with similar results. Water = water‐drinking mice. EtOH = alcohol‐consuming mice. *P < 0.05, **P < 0.01

NK cells, especially Ly49H^+^ NK cells, are the major effector cells responsible for eliminating virus‐infected cells at the early stage of MCMV infection. This process is dominated by Ly49H^+^ NK cell activation through Ly49H recognition of the viral protein m157. Therefore, the antiviral response is characterized by rapid expansion of Ly49H^+^ NK cells. We next determined how alcohol consumption affects the dynamic changes of Ly49H^+^ NK cells. Results indicated that alcohol consumption significantly decreased the number of NK cells and Ly49H^+^ NK cells in the spleen and liver at 1.5 and 3 days pi (Figure 5A, B, D, E). NK cells and Ly49H^+^ NK cells reached their peak on 5 days pi in the spleen and liver (Figure 5A, B, D, E). The number of splenic Ly49H^+^ NK and total NK cells was same in the two groups of mice on day 5 pi, but the number of these cells decreased quickly on day 6 pi in alcohol‐consuming mice compared to water‐drinking mice in which the number of NK cells and Ly49H^+^ NK cells did not change between day 5 and day 6 pi (Figure 5A, B). The number of liver NK cells decreased rapidly in both groups of mice on day 6 pi. There was no difference in the total NK and Ly49H^+^ NK cells in the liver between the two groups of mice on day 5 and 6 pi (Figure 5D, E). KLRG1 is an NK cell activation marker and expressed on Ly49H^+^ NK cells during MCMV infection.^46^ The pattern of dynamic change in KLRG1^+^ NK cells in the spleen and liver was same as the pattern of Ly49H^+^ NK cells in the respective organs (Figure 5C, F). The only difference was that the decline of splenic KLRG1^+^ NK cells in alcohol‐consuming mice was more pronounced on day 6 pi, where the number of these cells in alcohol‐consuming mice was significantly lower than in water‐drinking mice (Figure 5C).

### 3.6 Chronic alcohol consumption impairs NK cell maturation during MCMV infection

**Figure 6:**
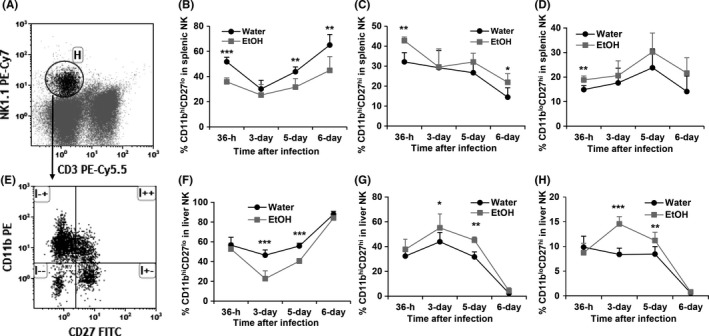
Effects of chronic alcohol consumption on NK cell maturation in the spleen and liver during the acute phase of MCMV infection. A, Dot plot shows gated NK (Gate H) cells in splenocytes. B, Percentage of CD11b^hi^CD27^lo^ cells in splenic NK cells. C, Percentage of CD11b^hi^CD27^hi^ cells in splenic NK cells. D, Percentage of CD11b^lo^CD27^lo^ cells in splenic NK cells. E, Dot plot shows CD11b^hi^CD27^lo^ (upper left quadrant), CD11b^hi^CD27^hi^ (upper right quadrant), CD11b^lo^CD27^hi^ (lower right quadrant), and CD11b^lo^CD27^lo^ (lower left quadrant) in gated splenic NK cells. F, Percentage of CD11b^hi^CD27^lo^ cells in liver NK cells. G, Percentage of CD11b^hi^CD27^hi^ cells in liver NK cells. H, Percentage of CD11b^lo^CD27^hi^ cells in liver NK cells. Data were analyzed by two‐way ANOVA with uncorrected Fisher's LSD test. Data = mean ± SD. Each group contained five mice in each independent experiment. Results are a representative of at least two biologically independent experiments with similar results. Water = water‐drinking mice. EtOH = alcohol‐consuming mice. *P < 0.05, **P < 0.01, ***P < 0.001

Based on cell surface marker CD27 and CD11b expression, the development and maturation of NK cells can be divided into four developmental stages. The phenotypes of the developmental stages from immature to mature are: CD27^lo^CD11b^lo^, CD27^hi^CD11b^lo^, CD27^hi^CD11b^hi^, and CD27^lo^CD11b^hi^.^47^ Acute MCMV infection enhances NK cell maturation to eliminate virally infected cells.^15^ We next determined how alcohol consumption affected NK cell maturation in the spleen and liver during MCMV infection. Figure 6A, E are dot plots showing gated NK cells and CD11b CD27‐defined NK subsets in gated NK cells, respectively. Chronic alcohol consumption significantly decreased the percentage of CD27^lo^CD11b^hi^ mature NK cells in the spleen at 36 hours, 5 days, and 6 days pi (Figure 6B), but increased the percentage of CD27^hi^CD11b^hi^ NK cells at 36 hours and 6 days pi (Figure 6C). Alcohol consumption significantly increased CD27^hi^CD11b^lo^ immature NK cells in the spleen at 36 hours pi (Figure 6D). In the liver, chronic alcohol significantly decreased mature CD27^lo^CD11b^hi^ NK cells (Figure 6F), increased CD27^hi^CD11b^hi^ (Figure 6G), and CD27^hi^CD11b^lo^ immature NK cells (Figure 6H) on day 3 and day 5 pi. Collectively, chronic alcohol consumption impairs NK cell maturation during the acute phase of MCMV infection.

### 3.7 Chronic alcohol consumption enhances NK cells activation and granzyme B production

**Figure 7:**
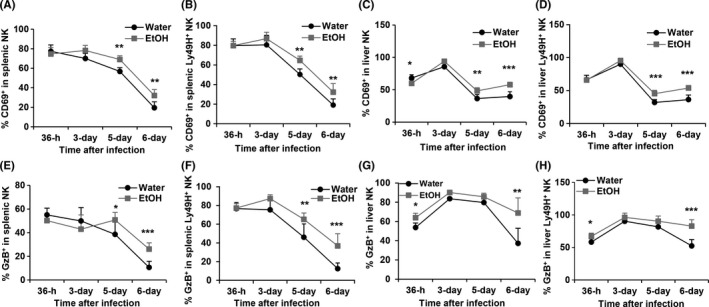
Effects of chronic alcohol consumption on the dynamic changes of the expression of CD69 and GzB in spleen and liver NK cells during the acute phase of MCMV infection. A, Percentage of CD69^+^ NK cells in splenic NK cells. B, Percentage of CD69^+^ NK cells in splenic Ly49H^+^ NK cells. C, Percentage of CD69^+^ NK cells in liver NK cells. D, Percentage of CD69^+^ NK cells in liver Ly49H^+^ NK cells. E, Percentage of GzB^+^ NK cells in splenic NK cells. F, Percentage of GzB^+^ NK cells in splenic Ly49H^+^ NK cells. G, Percentage of GzB^+^ NK cells in liver NK cells. H, Percentage of GzB^+^ NK cells in liver Ly49H^+^ NK cells. Data were analyzed by two‐way ANOVA with uncorrected Fisher's LSD test. Data = mean ± SD. Each group contained five mice in each independent experiment. Results are a representative of at least two biologically independent experiments with similar results. Water = water‐drinking mice. EtOH = alcohol‐consuming mice. *P < 0.05, **P < 0.01, ***P < 0.001

Viral infection activates NK cells, which is reflected by the upregulation of early activation marker CD69 expression and the production of effector molecule GzB. The duration and magnitude of NK cell activation are determined by the viral load. We next determined how alcohol consumption affects NK cell activation and GzB production in the spleen and liver. In the spleen, CD69 expression on NK cells peaked at 36 hours pi in the two groups of mice. In water‐drinking mice CD69 expression in NK and Ly49H^+^ NK cells progressively decreased from 36 hours through day 6 pi (Figure 7A, B). In alcohol‐consuming mice CD69 expression in NK cells was stable or slightly increased from 36 hours to 3 days pi, then decreased progressively from day 3 through day 6 pi (Figure 7A, B). The percentage of CD69^+^ NK cells and Ly49H^+^ NK cells in alcohol‐consuming mice was significantly higher than in their water‐drinking counterparts on day 5 and day 6 pi (Figure 7A, B). CD69 expression in liver NK and Ly49H^+^ NK cells peaked at 3 days pi, there was no difference between the two groups of mice (Figure 7C, D). The percentage of CD69^+^ NK and CD69^+^Ly49H^+^ NK cells decreased from day 3 to day 6 in the liver. The percentage of CD69^+^ NK and CD69^+^Ly49H^+^ NK cells was higher in alcohol‐consuming mice than in water‐drinking mice on day 5 and day 6 pi in the liver (Figure 7C, D). There was no difference in the percentage of GzB^+^ NK cells in splenic NK cells between the two groups of mice at 36 hours and 3 days pi, but the percentage of these cells in alcohol‐consuming mice was higher than in water‐drinking mice on day 5 and day 6 pi (Figure 7E). The percentage of GzB^+^ Ly49H ^+^ NK cells in splenic Ly49H^+^ NK cells in alcohol‐consuming mice was significantly higher than in water‐drinking mice on day 5 and day 6 (Figure 7F). The percentage of GzB^+^ NK cells in liver NK and Ly49H+ NK cells was significantly higher at 36 hours and 6 days pi in alcohol‐consuming mice than in water‐drinking mice (Figure 7G, H). Collectively, alcohol consumption enhanced CD69^+^ expression in NK and Ly49H^+^ NK cells on day 5 and day 6 pi, and also increased GzB‐producing NK and Ly49H^+^ NK cells on day 6 pi.

### 3.8 Chronic alcohol consumption decreases perforin production in spleen and liver

**Figure 8:**
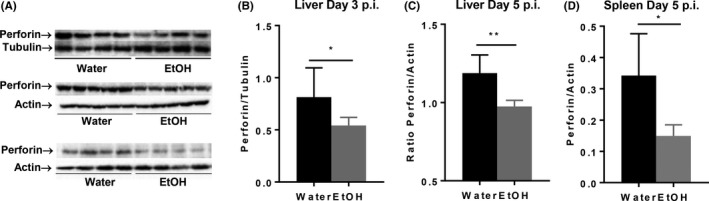
Chronic alcohol consumption inhibits the expression of perforin in liver and spleen during MCMV infection. A, Electrophoretogram of perforin in liver (top, 3 days pi, middle, 5 days pi) and spleen (bottom, 5 days pi), tubulin and actin were used as loading control. B, Ratio perforin to tubulin in liver after 3 days of infection. C, Ratio of perforin to actin in liver after 5 days of infection. D, Ratio of perforin to actin in spleen after 5 days of infection. Data were analyzed by two‐tailed, unpaired Student's *t* test. Data = mean ± SD. Each group contained 5 mice in each independent experiment. Results are a representative of at least two biologically independent experiments with similar results. Water = water‐drinking mice. EtOH = alcohol‐consuming mice. *P < 0.05, **P < 0.01

Perforin is the effector molecule that plays the critical role in the elimination of viral infected cells and in control of EMH development during MCMV infection.^38^ We used western blot to determine the expression of perforin in spleen and liver during MCMV infection. Results indicated that the expression of perforin in spleen and liver was significantly lower in alcohol‐consuming mice than in water‐drinking mice at 3 days and 5 days pi (Figure 8).

### 3.9 *Chronic alcohol consumption enhances CD8^+^ T‐cell activation during MCMV infection*

**Figure 9:**
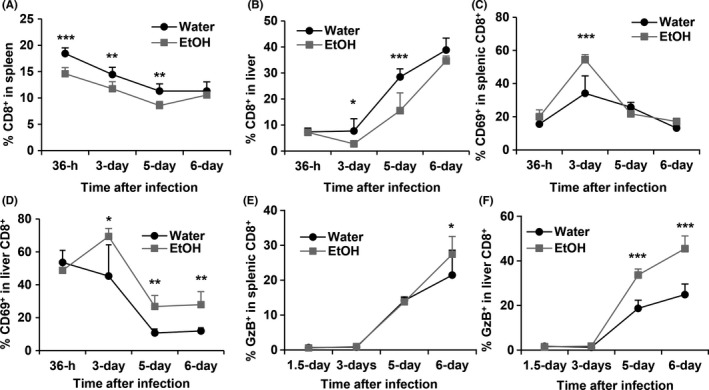
Effects of chronic alcohol consumption on CD8^+^ T cells during acute phase of MCMV infection. A, percentage of CD8^+^ T cells in splenocytes. B, Percentage of CD8^+^ T cells in liver leukocytes. C, Percentage of CD69^+^CD8^+^ cells in splenic CD8^+^ T cells. D, Percentage of CD69^+^CD8^+^ cells in liver CD8+ T cells. E, Percentage of GzB^+^ CD8^+^ cells in splenic CD8^+^ T cells. F, Percentage of GzB^+^ cells in liver CD8^+^ T cells. Data were analyzed by two‐way ANOVA with uncorrected Fisher's LSD test. Data = mean ± SD. Each group contained 4‐5 mice in each independent experiment. Results are a representative of at least two biologically independent experiments with similar results. Water = water‐drinking mice. EtOH = alcohol‐consuming mice. *P < 0.05, **P < 0.01, ***P < 0.001

CD8^+^ T cells play a key role in the final clearance of MCMV infection. We next determined how alcohol consumption affects CD8^+^ T‐cell response. Chronic alcohol consumption decreased the percentage of CD8^+^ T cells in spleen at 36 hours, 3 days, and 5 days but not 6 days after MCMV infection (Figure 9A). Alcohol consumption also led to a lower percentage of CD8^+^ T cells in liver but was only statistically significant on day 3 and day 5 after MCMV infection (Figure 9). The percentage of CD69^+^CD8^+^ T cells in splenic CD8^+^ T cells was higher in alcohol consuming mice than in water‐drinking mice on day 3 pi (Figure 9C). The percentage of liver CD69^+^CD8^+^ T cells was higher in alcohol consuming mice than in water‐drinking mice from day 3 through day 6 pi (Figure 9D). Alcohol consumption significantly increased the percentage of GzB^+^ CD8 ^+^ T cells in the spleen on day 6 pi (Figure 9E), and on day 5 and day 6 pi in the liver (Figure 9F). These results suggest that alcohol consumption decreases CD8^+^ T cells but enhances T‐cell activation during acute phase of MCMV infection.

## 4 DISCUSSION

In this study, our data clearly indicate that chronic alcohol consumption exacerbates MCMV infection and impairs viral clearance, which is evidenced by the increased viral load in spleen, and enhanced and prolonged body weight loss of alcohol‐consuming mice (Figure 1). The reduced blood IFN‐β level and decreased IFN‐γ‐ and GzB‐producing NK cells at 12 hours pi could facilitate the first round of viral replication and viral dissemination. Compromised Ly49H^+^ NK cell expansion and NK cell maturation could further impair viral clearance. The increased viral load would induce NK cell continuous activation and further induce a strong CD8^+^ T‐cell activation, which is reflected on the increase in CD69^+^, GzB^+^ NK cells and CD8^+^ T cells in the alcohol‐consuming mice. Compared to IFN‐γ and granzymes, perforin is the critical effector molecule that plays the decisive role in the elimination of virally infected cells and the clearance of viral infection. Results in this study have strongly implicated that impaired perforin production could be the major cause of alcohol exacerbation of MCMV infection. This notion is supported by the indirect evidence that alcohol inhibits splenomegaly, which is the hallmark of EMH, a biological process controlled by NK cells and perforin in MCMV infection,^38^ and by the direct evidence that the protein expression of perforin in spleen and liver was significantly down regulated in alcohol‐consuming mice (Figure 8).

During the acute phase of MCMV infection, initial innate immunity plays a crucial role in the control of the first round of viral replication in stromal cells and the dissemination of virus, which sets the baseline for the progression of infection.^22^ In an immunocompetent mouse, MCMV replication reaches its peak around 32 hours pi in spleen.^22^ MCMV infects spleen marginal zone stromal cells as early as 4‐6 hours pi and stimulates these cells to produce the first wave of IFN‐α/β through the activation of B cell‐mediated lymphotoxin (LT) αβ/LTβR/NF‐κB signaling pathway.^9^, ^22^, ^48^ The first wave IFN‐α/β, which may directly inhibit viral replication, will further activate NK cells to produce IFN‐γ. Deficiency in IFN‐α/β or NK/IFN‐γ dramatically enhances viral replication.^22^ The antiviral potency of IFN‐β is approximately 30 times stronger than IFN‐α.^13^ Chronic alcohol consumption significantly decreased blood level IFN‐β and the IFN‐γ‐producing NK cells at 12 hours pi (Figure 2), which would enhance MCMV replication and dissemination leading to the increase in viral load in the spleen as we observed 4 days pi (Figure 1). The decreased IFN‐γ‐producing NK cells could result from the decreased level of type I interferon because at this stage IFN‐α/β is the major cytokine to activate NK cells.^22^ The decrease in IFN‐γ‐producing NK cells could also result from the intrinsic defect in IFNAR signaling pathway in NK cells induced by alcohol. This issue needs to be further investigated.

While the first wave IFN‐α/β‐activated nonspecific NK cell activation plays a key role in the control of the first round of MCMV application, antigen‐specific NK cell activation dominates viral clearance. The antigen‐specific NK cell activation is featured by Ly49H^+^ NK cell proliferation and maturation.^21^ Alcohol consumption not only decreased Ly49H^+^ NK cell numbers in spleen and liver at 36 hours and 3 day pi (Figure 5), but also impaired NK cell maturation which was reflected in the decreased CD11b^hi^CD27^lo^ NK cells in spleen and liver (Figure 6). This deficiency in Ly49H^+^ NK cells would compromise viral clearance, which is consistent with the result that viral load was high at day 4 pi in the spleen of alcohol‐consuming mice (Figure 1A).

NK cell proliferation and maturation during MCMV infection are mainly driven by IL‐15 produced by DC.^11^ Alcohol consumption decreased IL‐15‐producing DC in the spleen and liver at 36 hours pi (Figure 4), which could result in decreased Ly49H^+^ mature NK cells (Figures 5 and 6). Engagement of Ly49H by m157 could compensate for reduction in NK cell proliferation and maturation due to reduced IL‐15 production.^49^ The number of Ly49H^+^ NK cells in spleen and liver of alcohol‐consuming mice was same as in the spleen and liver of water‐drinking mice at 5 and 6 days pi (Figure 5). This could result from the continuous engagement of Ly49H receptor by the increased m157 antigen to stimulate NK cell proliferation.

It is broadly accepted that alcohol is an immune suppressant. Surprisingly, during the acute phase of MCMV infection alcohol consumption continuously enhanced NK cell activation, which was reflected on the increased CD69 and granzyme B expression in NK cells, specifically Ly49H^+^ NK cells, at 5 and 6 days pi (Figure 7). This could be a compensatory activation of NK cells by the increased viral load. This phenomenon was also observed in immunocompetent C57BL/6 mice infected with m157‐mutated MCMV.^15^ Because of the inability of the immune system to clear viral infection, the increased viral load will continuously activate the immune system to induce inflammation. The inflammatory cytokines increase the expression of activation markers CD69 and GzB in NK cells.^15^

IFN‐γ and perforin are the two most important effector molecules that control viral replication and clearance.^12^, ^13^ IFN‐γ plays a key role in the control of viral replication.^12^ Perforin plays the critical role in the clearance of virally infected cells.^13^ Although granzymes, such as GzB, also play important roles in the elimination of virally infected cells, compared to perforin, the effects of granzymes in MCMV infection are dispensable, which is evidenced by the fact that granzyme KO mice can survive MCMV infection, while perforin KO mice cannot survive MCMV infection.^50^ One of the major reasons why alcohol‐consuming mice show reduced MCMV clearance is the defect in perforin production. This conclusion is based on the following three lines of evidence: (a) Inhibited splenomegaly and prolonged body weight loss in alcohol‐consuming mice. MCMV‐induced EMH is controlled by NK cells, especially perforin produced by NK cells.^38^ Body weight loss is induced by inflammation, which is associated with viral infection. Prolonged body weight loss reflected a failed clearance of virally infected cells; (b) Enhanced GzB^+^ NK cells in alcohol‐consuming mice; (c) Western blot results directly indicated that the protein expression of perforin was decreased in the liver and spleen of MCMV‐infected alcohol‐consuming mice (Figure 8).

NK cells and CD8^+^ T cells are the two most important types of effector cells that control MCMV infection. Accumulating evidence supports that these two types of cells have a reverse correlation in MCMV infection: a strong NK cell response leads to a weak CD8^+^ T‐cell response, compromises CD8^+^ T‐cell memory formation and facilitates the latency of the virus; a weak NK cell response induces a strong CD8^+^ T‐cell activation and enhances viral clearance by CD8^+^ T cells.^15^, ^16^ Indeed, in this study, we found that alcohol consumption enhanced CD8^+^ T‐cell activation which was featured by the enhanced expression of CD69 and GzB in CD8^+^ T cells (Figure 9). However, how the impaired NK cell response affects CD8^+^ T‐cell memory and the latency of the MCMV in alcohol‐consuming mice need to be investigated further, because alcohol consumption itself can directly impair CD8^+^ T‐cell response in the steady state and during bacterial or viral infection.^25^, ^51^, ^52^

In summary, chronic alcohol consumption impairs type I interferon production at the early stages of acute MCMV infection, which compromises the nonspecific NK cell activation. Alcohol consumption further impairs IL‐15 production of DC, which leads to dysregulation of Ly49H‐specific NK cell activation reflected in the compromised Ly49H^+^ NK cell maturation, proliferation and perforin production. The dysfunction of nonspecific and specific NK cell activation results in increased viral load, inhibited splenomegaly and prolonged body weight loss. The increased viral load leads to continuous inflammation to enhance NK and CD8^+^ T‐cell activation. Therefore, targeting type I interferon and IL‐15 signaling pathways could be the possible therapeutic approaches for the recovering antiviral immune response of CMV infection in alcoholics.

## DISCLOSURES

The authors have no financial conflicts of interest.

## AUTHOR CONTRIBUTIONS

H. Zhang conceived the project and designed the study and experiments; A. Little, Y. Li, F. Zhang, and H. Zhang performed research; H. Zhang; A. Little and Y. Li analyzed data; H. Zhang and A. Little wrote the paper.
